# Integrated Flame Weeding and Biochar Reutilization Enhance Soybean Yield by Promoting Soil Microbial Abundance

**DOI:** 10.3390/plants15203132

**Published:** 2026-10-14

**Authors:** Ziyang Qu, Hongbin Cheng, Qiyun Xu, Mengru Su, Mingniang Qi, Meishuang Li, Yongkang Chen, Chunping Jian, Yongpeng Wang, Xiaorong Fan

**Affiliations:** 1 Sanya Institute of Nanjing Agricultural University, College of Resources and Environmental Sciences, Nanjing Agricultural University, Nanjing 210095, China; 2 Hainan State Farms Academy of Sciences Group Co., Ltd., Sanya 572024, China

**Keywords:** biochar, flame weeding, foliar spraying, physiological improvement, microbial communities, root nodulation, soil properties, soybean

## Abstract

Weed interference and crop residue are major constraints on sustainable soybean production, particularly in intensive tropical agriculture. Flame weeding provides a non-chemical approach for weed management, but the agronomic value of carbonized residues generated during this process remains largely unexplored. In this study, flame-weeding temperatures ranging from 300 to 800 °C were first screened based on weed carbonization efficiency and air-quality condition, after which 500 and 600 °C were selected for field evaluation. A two-season field experiment was conducted using a 3 × 2 treatment structure combining three pre-sowing flame-weeding temperatures (0, 500, and 600 °C) with or without biochar from flame-weeding (BFW) foliar spraying. Each treatment included four replicates. Flame weeding was applied before soybean sowing, whereas BFW foliar spraying was performed at the V4, R2, R4, and R6 growth stages. Flame weeding altered the soil environment, increasing electrical conductivity and reducing soil nematode abundance. Combined treatments produced the highest yields in soybean. In particular, T600-BFW increased the yield per plant by 73.32% and 32.43% relative to the CK in the two seasons, respectively. Flame weeding exerted a significant lethal effect on soil nematodes. Soil nematode density declined from 149.75 to 2.92 ind·20 g^−1^ of soil at week 10 following T600 treatment. Compared to CK, T600-BFW increased the root nodule number by 31.71%. The quantities of bacteria, fungi, and actinomycetes in T500 and T600 increased significantly after flame weeding. Redundancy analysis identified soil temperature, electrical conductivity, and pH as major variables associated with microbial community variation. The PLS-PM model showed that soil microorganisms exerted a significant positive effect on soybean yield. These findings indicate that flame weeding and BFW reutilization enhance soybean yield through complementary soil–microbe–root and leaf physiological improvement. This integrated strategy provides a potential pathway for simultaneously managing weeds, reutilizing field-derived carbonized biomass, and improving soybean production.

## 1. Introduction

Weed interference and straw residue are critical constraints on the development of green soybean production [1,2]. Uncontrolled weed interference can reduce soybean yields by 52.1% in North America, resulting in economic losses exceeding $1.7 billion [3]. In China, over one billion tons of crop straw are generated annually, the majority of which remains inadequately utilized [4]. In response to the ban on straw burning, tropical agricultural regions such as Hainan must invest substantial labor and material resources in managing agricultural wastes, including straw and weeds left over after the previous harvest [5]. This not only shortens the time available for crop rotation but also adversely affects subsequent soybean production. Therefore, environmentally sound approaches are required to address the dual constraints of weed pressure and crop residue persistence in tropical agriculture.

Flame weeding is an effective mean of weed control and straw consumption. It acts through short-duration high temperatures to disrupt the cell membrane and protoplasmic structure of plants, causing tissue dehydration and protein denaturation and ultimately leading to the loss of regenerative capacity [6]. Flame weeding was applied to tomato and cabbage crops in 2002 and 2003. The results showed that flame weeding effectively reduced the incidence of blossom end rot in tomato and provided weed-control efficacy comparable to that of herbicides [7]. A study of flame weeding applied at different times of the day revealed that weeds were significantly less tolerant to thermal exposure at 8 h after sunrise, attributable to their lower relative water content, with post-treatment damage exceeding 76% [8]. Soybean yield parameters showed no significant differences across varying flame-weeding doses, flame heights, or growth stages. Nevertheless, application during the vegetative growth phase resulted in substantially greater plant injury, suggesting that the flowering stage represents a more suitable timing for flame weeding [6,9].

Biochar is a carbon-rich material primarily derived from agricultural waste feedstocks through high-temperature decomposition at 300–1000 °C under oxygen-limited or anaerobic conditions [10]. Different biomass feedstocks and pyrolysis temperatures result in variations in the physical structure and elemental composition of biochar [11,12,13]. Previous studies have shown that biochar yield decreases with increasing pyrolysis temperature because more carbon is released as gaseous products at elevated temperatures, including CO, CO_2_, and CH_4_ [14,15]. A relatively low pyrolysis temperature (350 °C) is suitable for biochar production, as nutrient losses are significantly lower when rice straw is converted into biochar [16]. As the pyrolysis temperature was increased from 600 to 1000 °C, the content of CO, CO_2_, and CH_4_ together accounted for more than 86% of the gaseous products. The proportion of CO and CH_4_ increased with temperature, while that of CO_2_ decreased [12].

Recycling agricultural waste into biochar is a beneficial practice for promoting sustainable agricultural development. In previous studies, biochar had been prepared from agricultural residues such as rice [17], wheat [18], soybean [19], corn [20], peanut [21], and canola [21] for use in soil remediation and wastewater treatment. Biochar, when used as a soil amendment, can decrease the availability of heavy metals, promote plant growth, and enhance plant disease resistance. Compared with control, the availability of Cu and Cd in the biochar amended soils decreased by 17–31% and 3–17%, respectively [22]. A three-year field experiment revealed that the incorporation of rice straw-derived biochar into fields continually increased rice yield by 8.0%, 1.6%, and 7.3% [23]. Plant roots tend to proliferate in biochar-amended soil, where biochar can stimulate root activity, increase the number of root tips, and expand the rhizosphere zone, thereby enhancing plant uptake of nitrogen (N) and phosphorus (P) [24,25,26]. The relatively higher pH of rice-straw-derived biochar, along with its lower contents of available nitrogen and dissolved organic carbon helps reduce nitrous oxide emissions after nitrogen fertilizer application in farmland [27]. Biochar can serve as a shelter for microbial colonization, which enhance the abundance of rhizobacteria capable of transforming organic sulfur (S) and P into bioavailable forms [28,29]. Upon application to soil, biochar can enhance microbial diversity in the crop rhizosphere, promote root nodulation in crops such as soybean, and consequently increase crop yield [30].

Although the role of biochar as a soil amendment has been extensively documented in the literature, studies on the foliar application of biochar remain relatively scarce. Extracts of both wheat and maize straw biochar significantly increased cabbage yield, vitamin C content, and soluble protein content while decreasing nitrate content at 50- or 100-fold dilutions [31]. Foliar application of the highest biochar and sulfur treatment (500 mg L^−1^ biochar + 4.5 g L^−1^ sulfur) increased total bulb yield by 32.64–35.13% and mean bulb weight by 29.30–29.66% across two growing seasons [32]. In addition, spraying 3% nano-biochar colloid solution can effectively increase the plant height, plant biomass, fruit set and single-fruit weight of tomato plants, helping tomatoes resist salt stress [33].

The present study had two primary objectives. First, to identify an alternative farming practice to conventional straw burning, we aim to employ flame-weeding technology to eliminate agricultural waste while simultaneously improving soil properties. Second, to further improve production efficiency, we actively utilize the carbon-based materials generated from flame weeding to produce environmentally friendly biochar materials, thereby achieving promotion of both shoot and root growth. We combined flame weeding with foliar spraying of biochar from flame weeding (BFW) to investigate the effects of these two practices on tropical soil properties, soybean growth and development, yield, and seed quality. This study provides a basis for developing efficient and sustainable soybean production systems in tropical regions.

## 2. Materials and Methods

### 2.1. Experimental Site and Ecological Conditions

The field experiment was conducted over two seasons. The first season was conducted in the summer of 2025 at the Batou Base, Yazhou Bay Science and Technology City, Sanya, Hainan, China, whereas the second season was conducted in the winter of 2025 at the National Modern Agricultural Industrial Park, Sanya, Hainan, China. The experimental site was located within the Agricultural Experimental Zone of Yazhou Bay Science and Technology City, a typical tropical monsoon climate region characterized by abundant year-round heat, sufficient solar radiation, and relatively stable diurnal temperature fluctuations. These conditions were suitable for multi-season soybean cultivation and field studies. The soil type was red-yellow podzolic soil. The plow layer was relatively loose and well drained, with moderate water- and nutrient-holding capacities. To characterize the initial soil conditions, composite soil samples were collected from the 0–20 cm soil layer before sowing using a five-point sampling method. The basic physicochemical properties were analyzed to serve as baseline data for subsequent assessments of soil environmental changes and crop responses.

### 2.2. Experimental Material

The soybean cultivar ‘Nannong 99-6’ was provided by Nanjing Agricultural University. The biochar used in this study was obtained from carbonized plant residues generated during flame weeding conducted at 500 °C. The collected biochar was ground using a vibratory mill and passed through a 300-mesh sieve to obtain initial carbon powder with a relatively uniform particle size. The basic properties of biochar from flame weeding are detailed in Table 1. A suspension was prepared by mixing 75 g of biochar powder to 30 L of deionized water. The suspension was circulated using a stainless-steel jet self-priming pump (Guangzhou Lingxiao Pump Co., Ltd., Guangzhou, China). The combined effect of oxidative etching, bubbling dispersion, and mechanical shear further reduced the particle size and produced a relatively stable aqueous dispersion. After circulation, the mixture was allowed to settle, and the supernatant was collected as the raw biochar dispersion. The foliar spray material (biochar from flame weeding, BFW) was prepared by diluting the dispersion 300-fold with water before application.

### 2.3. Experimental Design and Details

This experiment adopted a two-factor factorial design, with flame-weeding temperature and foliar spraying as fixed factors. The design comprised six treatments, each with four replicates (Table 2). The plot size was 200 cm × 180 cm. The treatments were as follows: CK (no flame weeding and no foliar spraying), BFW (no flame weeding but sprayed with a 300-fold dilution of BFW), T500 (flame weeding at 500 °C but no spraying), T500-BFW (flame weeding at 500 °C with spraying of a 300-fold dilution of BFW), T600 (flame weeding at 600 °C and no spraying), and T600-BFW (flame weeding at 600 °C with spraying of a 300-fold dilution of BFW). For all flame-weeding treatments, the burning duration was standardized at 2 min to ensure comparability among temperature treatments. Foliar spraying was conducted four times during the V4 (vegetative stage), R2 (initial flowering), R4 (pod formation), and R6 (grain filling) stages.

An infrared thermometer (Suzhou Tasi Electronic Industrial Co., Ltd., Suzhou, China) was used for temperature measurement. The instrument had a measurement range of −50 to 880 °C and a 650 nm laser pointer for target alignment. The laser aiming spot was positioned approximately 5–10 cm beyond the visible flame boundary. The distance between the thermometer and the measurement target was approximately 50–70 cm. Instrument readings were recorded at 60 s intervals during each application. Temperature fluctuations may occur due to air flow and manual movement during actual use. For this reason, an infrared thermometer was used to measure the temperature at the above-mentioned positions, and the gas cylinder valve was adopted to control the flame jet intensity. Once the temperature reaches the required standard, the weeds within the designated plot are subjected to incineration treatment.

### 2.4. Physical and Chemical Parameters of Soil

To characterize temporal changes in soil conditions associated with flame-weeding treatments, pre-sowing monitoring was performed at 2-week intervals, yielding seven measurements in total. Post-sowing assessments were conducted before foliar spraying and at harvest. The measured parameters included soil moisture content (SMC), soil temperature (ST), electrical conductivity (EC), pH, and redox potential (Eh). SMC, ST, EC, and pH were measured in situ using soil sensors (Shandong Saien Electronic Technology Co., Ltd., Shandong, China), whereas Eh was measured using a portable oxidation–reduction potential (ORP) meter (Clean Instruments Co., Ltd., Shanghai, China).

### 2.5. Soil Biological Traits

Soil nematodes, bacteria, actinomycetes, and fungi were monitored from 0 to 12 weeks before sowing. Bacterial, actinomycete, and fungal populations were determined using the dilution plate-count method throughout the soybean growing season, with results expressed as colony-forming units (CFU) per gram of dry soil. Soil nematodes were extracted and counted using sucrose centrifugation, and their abundance was expressed as individuals per 20 g of dry soil.

### 2.6. Photosynthetic Traits

Net photosynthetic rate, transpiration rate, intercellular CO_2_ concentration, and stomatal conductance of functional leaves were measured using the Li-6800 portable photosynthesis system (LI-COR, Inc., Lincoln, NE, USA) before the first spray and after the first, second, third, and fourth sprays, as well as at harvest. Measurements were conducted under a photosynthetic photon flux density of 1200 μmol m^−2^ s^−1^, CO_2_ concentration of 400 μmol mol^−1^, leaf chamber temperature of 26 °C, and relative humidity of 23% during clear, windless days between 9:00 and 11:00. For each plot, the third youngest fully expanded leaf from the main stem of uniformly growing plants was selected for measurement. Meanwhile, the chlorophyll relative content was determined using a SPAD meter, and the leaf area (LA) was measured with a leaf area meter.

### 2.7. Agronomic Traits and Root Morphology Parameters

The dynamic changes in plant height (PH) and leaf area (LA) were measured at different growth stages of the soybean, and the pod height on the main stem (BPH), number of main stem nodes (NNMS), number of branches (BN), stem diameter (SD), root length (RL), lateral root number (LRN), and nodule number (NN) were measured at the harvest stage. PH, BPH, and RL were measured using a measuring tape, while SD was measured with a digital vernier caliper. The NNMS, BN, LRN, and NN were counted manually.

### 2.8. Biomass Accumulation and Allocation Parameters

At harvest, plants were separated into roots, stems, leaves, pods, and seeds. The fresh weights of each component were recorded individually. The shoot fresh weight and whole-plant fresh weight were calculated. The samples were first oven-dried at 105 °C for 30 min and subsequently dried at 75 °C to a constant weight. Dry weights of roots, stems, leaves, pods, and seeds were determined, and the shoot dry weight, whole-plant dry weight, and root-to-shoot ratio (R/S) were calculated.

### 2.9. Yield and Yield Components

The number of pods per plant (PPP), number of seeds per plant (SPP), number of pods in each pod category, the number of chaff pods, 100-seed weight (100-SW), the seed length (SL), and seed width (SWD) were measured for each plant. The 100-seed weight and seed dimensions were determined using an intelligent seed-counting system (Zhejiang Top Cloud-Agri Technology Co., Ltd., Hangzhou, China), whereas the other traits were counted manually or statistically through manual sorting.

### 2.10. Nitrogen and Phosphorus Absorption and Grain Quality Parameters

Samples were ground to pass through a 100-mesh sieve. Total nitrogen (TN) and total phosphorus (TP) concentrations in shoot and seed samples were determined using a flow analyzer. Nitrogen and phosphorus accumulation were calculated based on the dry weight of the organs. Seed-quality traits were assessed using a near-infrared analyzer, including seed moisture content (GMS), protein content (PC), crude fat content (CFC), and water-soluble protein content (WSPC).

### 2.11. Soil Microbial DNA Extraction, High-Throughput Sequencing, and Data Processing

Soil samples were collected from each plot at harvest and immediately stored at −80 °C until DNA extraction. Total DNA was extracted from approximately 0.5 g of each soil sample using the E.Z.N.A.^®^ Soil DNA Kit (Omega Bio-tek, Norcross, GA, USA). DNA integrity was evaluated by 1% agarose gel electrophoresis, and the DNA concentration and purity were determined using a NanoDrop 2000 spectrophotometer (Thermo Fisher Scientific, Waltham, MA, USA). Bacterial communities were amplified using the V3–V4 region of the 16S rRNA gene with primers 338F/806R, whereas fungal communities were amplified using the ITS region with primers ITS1F/ITS2R. PCR reactions were performed in a final volume of 20 μL containing 4 μL of 5 × FastPfu Buffer,2 μL of 2.5 mM dNTPs, 0.8 μL each of forward and reverse primers, 0.4 μL of FastPfu DNA Polymerase, 10 ng of template DNA, and nuclease-free water to volume. The PCR amplification procedure is 95 °C for initial denaturation for 3 min; 27 cycles of denaturation at 95 °C for 30 s, annealing at 55 °C for 30 s, and extension at 72 °C for 45 s; followed by a final extension at 72 °C for 10 min. PCR amplicons were purified, quantified, mixed in equal molar ratio for library construction, and sequenced on the Illumina NovaSeq/NextSeq 2000 platform (Illumina, Inc., San Diego, CA, USA). Raw sequencing reads were quality-filtered and merged to generate high-quality sequences. The resulting sequences were clustered into operational taxonomic units (OTUs) and taxonomically annotated. Alpha diversity, beta diversity, and differentially abundant taxa were subsequently analyzed.

### 2.12. Data Analysis

All data were analyzed using two-way ANOVA to evaluate the effects of flame-weeding temperature, foliar spraying, and their interaction on measured variables. Statistical significance was set at *p* < 0.05, and post hoc multiple comparisons were performed using Tukey’s method. GraphPad Prism 10 and R language were used for chart creation, and statistical analysis was performed on the JMP 11 platform. Microbial community analyses included the Kruskal–Wallis rank-sum test, principal coordinate analysis (PCoA), analysis of similarities (ANOSIM), linear discriminant analysis effect size (LEfSe), redundancy analysis (RDA), and Mantel tests. These analyses were used to characterize microbial diversity and community composition, identify differentially abundant taxa, and evaluate associations between microbial communities and environmental variables. Partial least squares path modeling (PLS-PM) analysis was performed using the R language package plspm.

## 3. Results

### 3.1. The Optimal Carbonization Temperature for Flame Weeding

**Figure 1:**
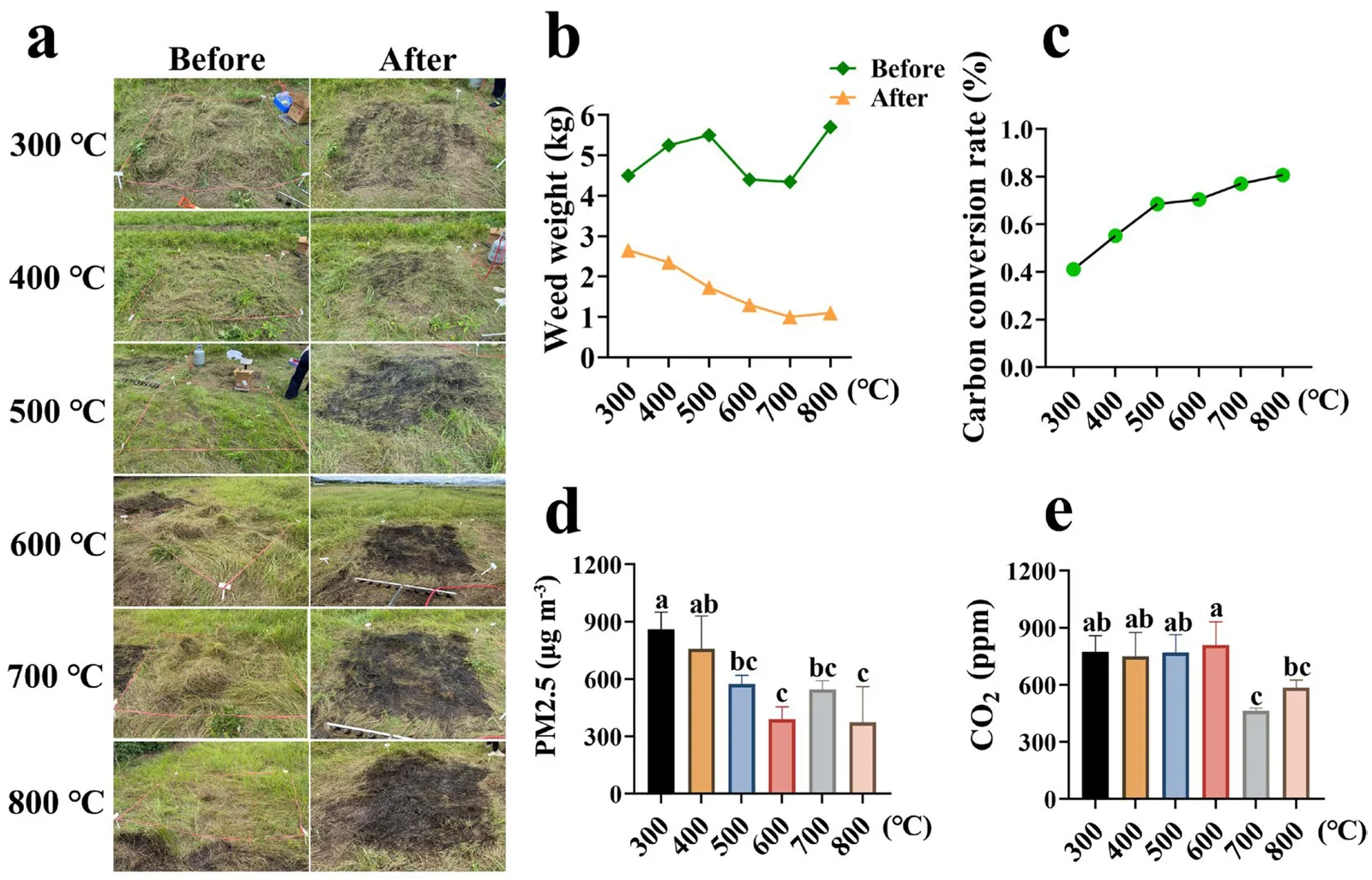
The optimal carbonization temperature for flame weeding. (**a**) Field photograph of burning weeds in farmland with a flame weeder. (**b**) Weed weight before and after flame-weeding treatment. (**c**) Carbonization rate at different flame-weeding temperatures. (**d**) PM2.5 concentrations at different flame-weeding temperatures. (**e**) CO_2_ concentrations at different flame-weeding temperatures. Means ± SDs with different letters in the figures indicate significant differences according to Tukey’s multiple range test (*p* < 0.05) among all treatments.

To determine the optimal flame temperature for effective flame weeding, this study established six flame temperature gradients and evaluated the effects of flame temperatures on weed carbonization and air quality. As shown in Figure 1a, different degrees of combustion performance were observed across the flame temperatures. With an initial weed biomass of 4.35–5.70 kg per plot, the carbon conversion rate increased from 41.11% to 80.70% with rising flame temperature (Figure 1b,c). At 500–600 °C, the carbon conversion rate had already reached 68.85 to 70.45%, indicating effective conversion of field residues. Compared with 300 °C, the PM2.5 concentrations were significantly reduced from 861.8 μg m^−3^ to 574.5 and 389 μg m^−3^ at 500 and 600 °C, respectively (Figure 1d). Similarly, compared with 300 °C (774.25 ppm), the atmospheric CO_2_ concentration at 700 and 800 °C significantly decreased to 464.75 and 585 ppm, reductions of 40.0% and 24.4%, respectively (Figure 1e).

### 3.2. The Effects of Flame Weeding on Soil Ecology

**Figure 2:**
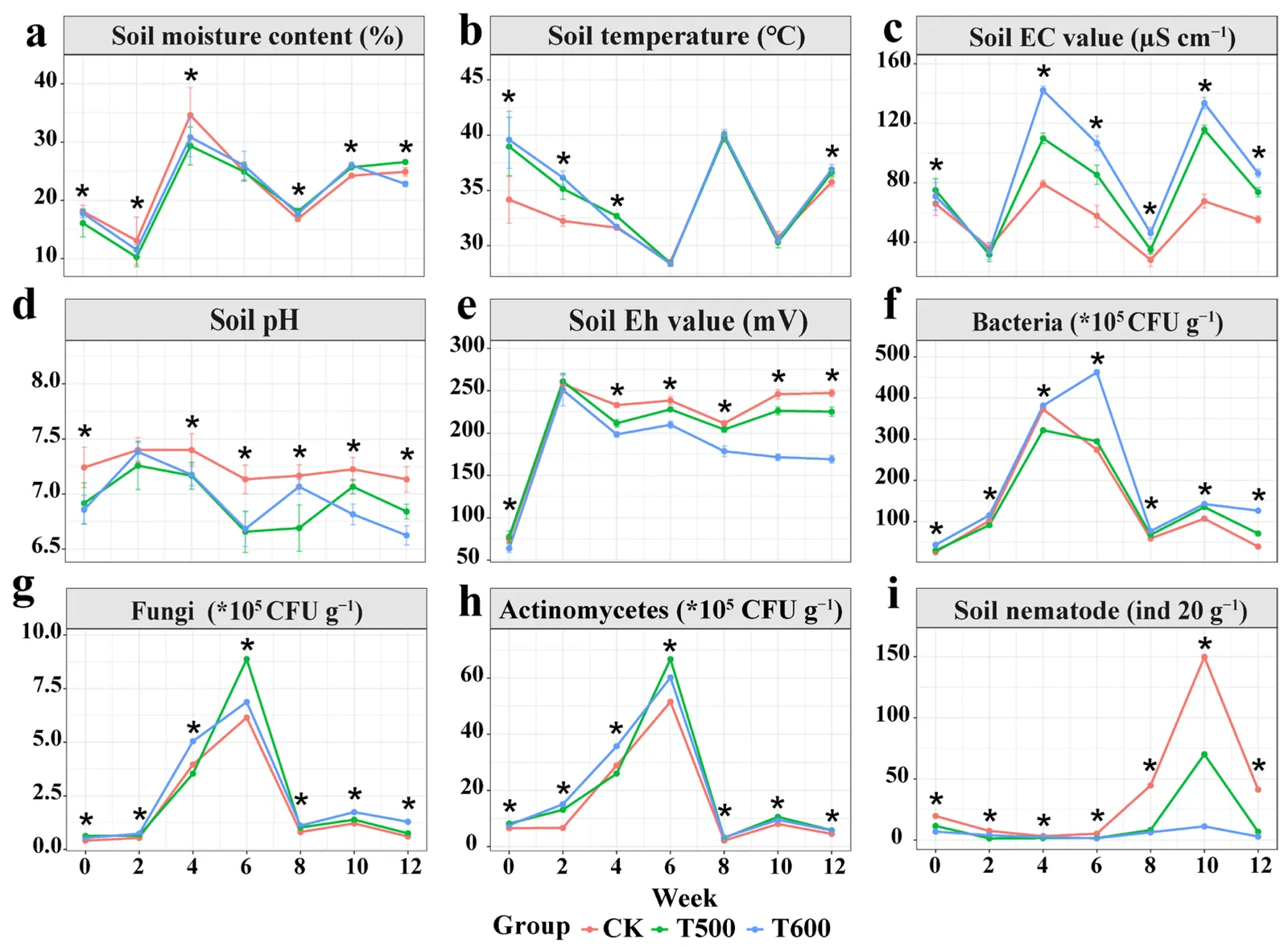
Temporal responses of soil physicochemical properties and soil biota to flame-weeding treatments during the 12-week pre-sowing period. (**a**) Soil moisture content; (**b**) soil temperature; (**c**) soil electrical conductivity (EC); (**d**) soil pH; (**e**) soil redox potential (Eh); (**f**) bacterial abundance; (**g**) fungal abundance; (**h**) actinomycete abundance; and (**i**) soil nematode abundance. CK, no flame weeding; T500 and T600, flame weeding at 500 and 600 °C, respectively. Values are means ± SDs. Asterisks indicate significant differences between flame-weeding treatments and CK at the corresponding sampling time according to Tukey’s multiple-comparison test (*p* < 0.05).

To elucidate the effects of flame weeding on soil ecology, we continuously monitored the soil physicochemical properties and soil biota for 12 weeks after flame weeding (Figure 2). Under identical soil parameters, both CK and treated soils exhibited similar temporal fluctuation patterns. However, different indicators displayed contrasting trends: for instance, soil moisture and temperature showed opposite trends, whereas the patterns of soil fungi and actinomycetes showed broadly similar patterns. At week 4, soil electrical conductivity (EC) in T500 and T600 significantly increased by 38.88% and 79.78% respectively, while soil pH fluctuated between 6.625 and 7.38 during the post-treatment period. Furthermore, flame weeding exerted a pronounced lethal effect on soil nematodes, with T600 maintaining a low density of 2.92 ind·20 g^−1^ by week 10, which was 98.1% lower than that in CK (149.75 ind·20 g^−1^).

### 3.3. Effects of the Combined Application of Biochar from Flame Weeding (BFW) on Soybean Yield

**Figure 3:**
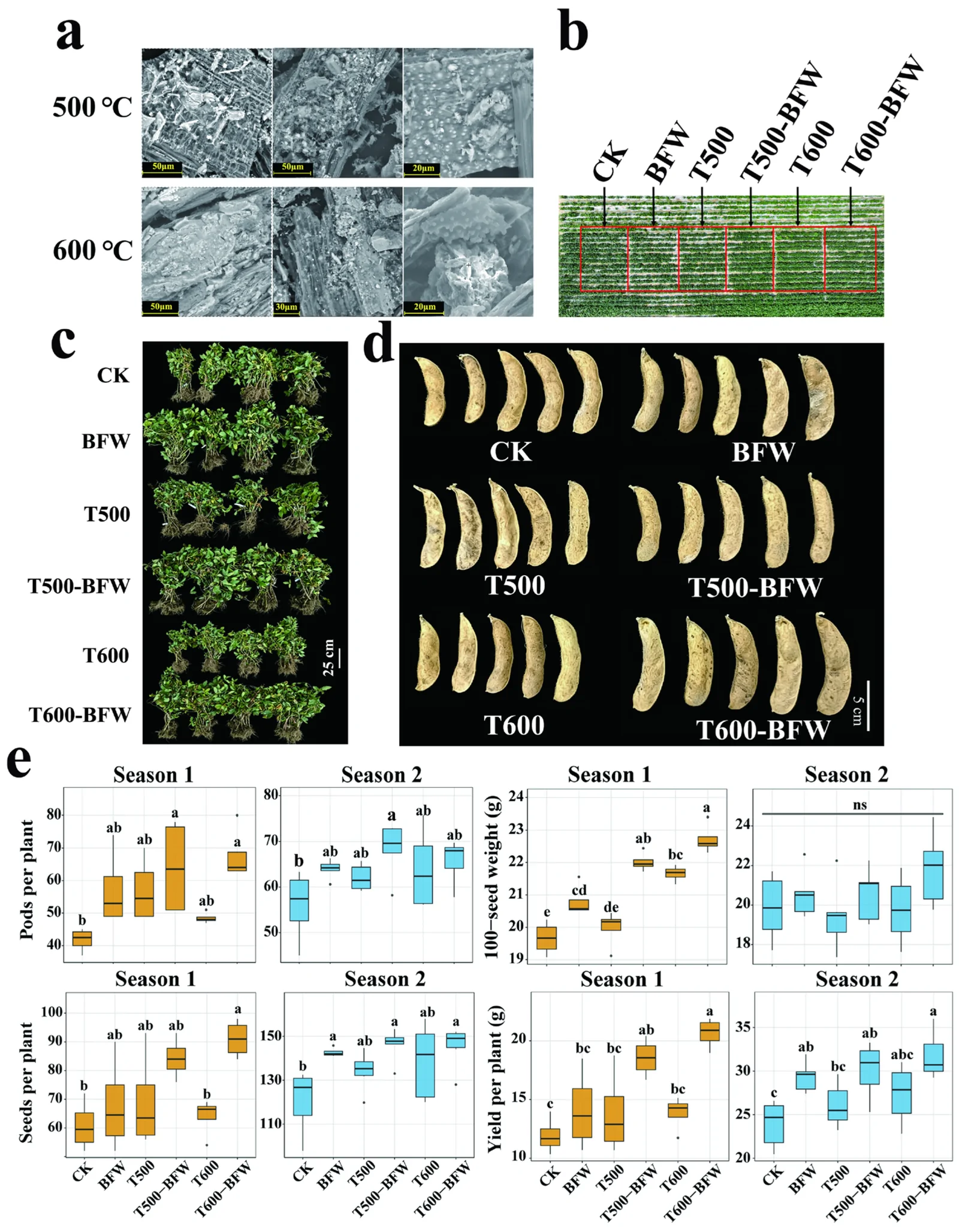
Effects of the application of flame weeding and BFW foliar spraying on soybean yield. (**a**) Surface morphology of BFW obtained at different temperatures observed by scanning electron microscopy (SEM). (**b**) Aerial photograph of field trials on flame weeding and combined application of BFW. (**c**) Phenotypic traits of soybean at the harvest stage under different treatments. (**d**) Pod morphology of soybean at the harvest stage under different treatments. (**e**) Yield indicators of soybean field experiments in the summer and winter of 2025. Means ± SDs with different letters in the figures indicate significant differences according to Tukey’s multiple range test (*p* < 0.05) among all treatments. ns means not significant.

Biochar from flame weeding (BFW) is a carbonized by-product generated during flame weeding. To clarify the effect of flame temperature on weed carbonization, the surface morphology of BFW obtained at different temperatures was observed using scanning electron microscopy (SEM) (Figure 3a). At flame temperatures of 500 and 600 °C, BFW retained plant vascular bundle-like microstructures, whereas the tissues transformed into lamellar and porous structures as the temperature increased. Energy-dispersive X-ray spectroscopy (EDS) analysis revealed that BFW samples produced at different flame-weeding temperatures consisted primarily of carbon (C), oxygen (O), silicon (Si), and other mineral elements. The proportion of carbon ranged from 33.99% to 35.99%, and that of potassium ranged from 3.05% to 3.21%. No significant differences in elemental composition were observed among BFW samples at different flame-weeding temperatures (Table 1). Accordingly, BFW generated at 500 °C was selected to prepare foliar spray suspension. Across two field seasons, both flame-weeding treatments at 500 °C or 600 °C and foliar application of BFW improved soybean yield-relative traits to varying extents. The combined treatments produced greater yield enhancement than flame weeding alone and CK (Figure 3b,d). Compared to CK, T600-BFW produced the highest yield per plant in both seasons, with significant increases of 73.32% and 32.43%, respectively. T500-BFW ranked second in yield per plant, whereas T500 showed the smallest increase (Figure 3e). In the first season, T600-BFW exhibited the highest 100-seed weight and seeds per plant, which were 15.56% and 49.79% higher than those of CK, respectively. In the second season, no significant differences in 100-seed weights were observed among the treatments, whereas the seeds per plant of T600-BFW was increased by 20.38% compared with that of CK (Figure 3e). Regarding pods per plant, all flame-weeding and BFW-application treatments showed significantly higher values than CK, whereas no significant differences were observed between the flame-only and foliar-application treatments.

To investigate the interaction between flame weeding and BFW foliar spraying, yield-related traits were analyzed using two-way ANOVA (Table S1). The results showed that both flame weeding and BFW foliar spraying significantly improved yield, with the foliar spraying exerting a more pronounced effect (*F*-value = 22.4229 and 24.7718 in the first and second seasons, respectively). However, the interaction between flame weeding and BFW foliar spraying was not significant.

### 3.4. Effects of the Combined Application of BFW on Growth Traits of Soybean

**Figure 4:**
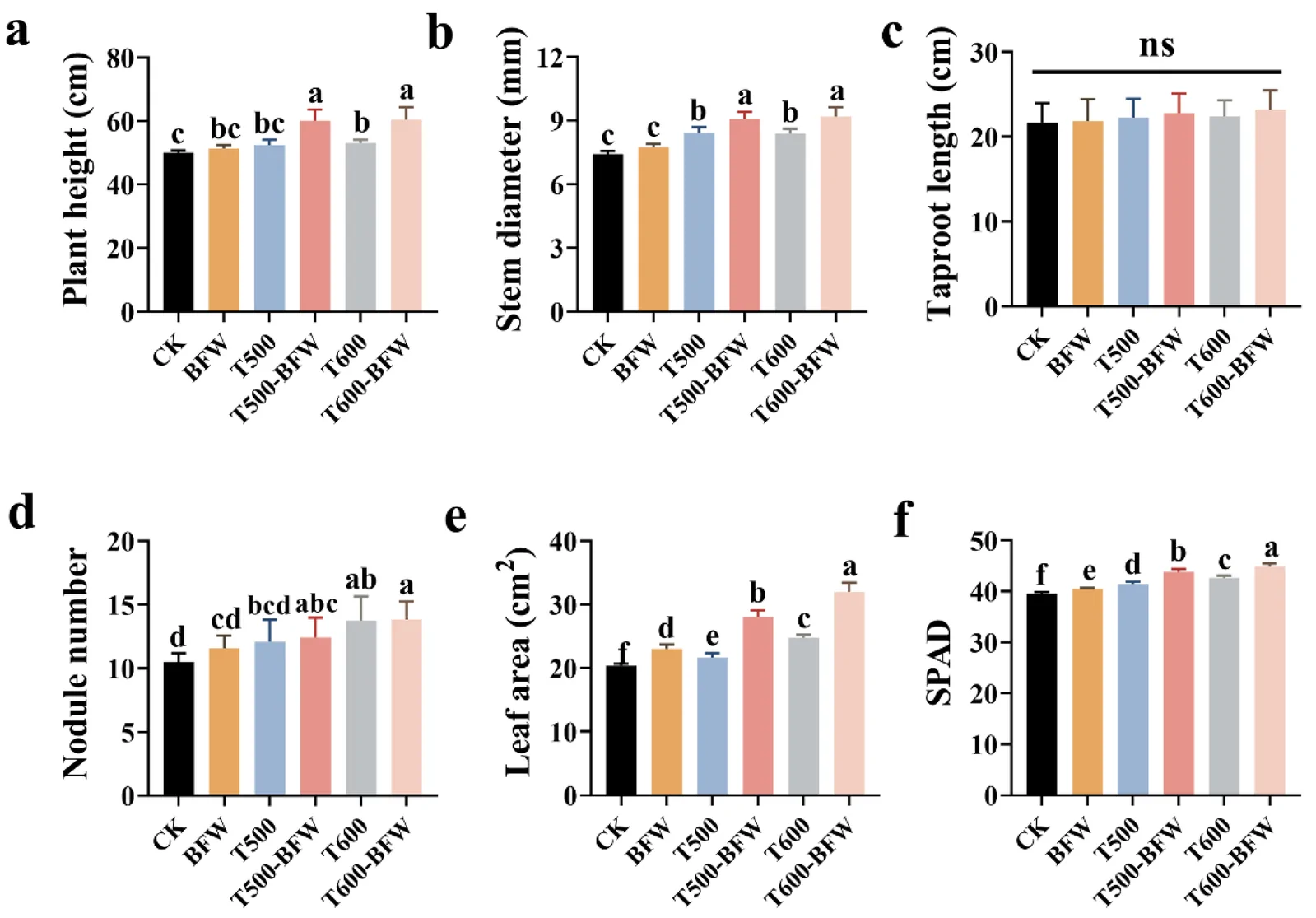
Effects of flame weeding and BFW foliar spraying on soybean growth and root traits at harvest. (**a**) Plant height; (**b**) stem diameter; (**c**) taproot length; (**d**) nodule number; (**e**) leaf area; and (**f**) SPAD value. CK, untreated control; BFW, foliar application of biochar derived from flame weeding; T500 and T600, flame-weeding treatments at 500 and 600 °C, respectively; T500-BFW and T600-BFW, corresponding combined treatments. Values are means ± SDs. Different lowercase letters indicate significant differences among treatments according to Tukey’s multiple-comparison test (*p* < 0.05); ns indicates no significant difference.

To further elucidate the effects of flame weeding and BFW foliar spraying on soybean growth, nutrient accumulation, and seed quality, the corresponding traits were measured at harvest (Figure 4). Different treatments exerted significant influences on plant morphogenesis. In particular, plant height and stem diameter showed similar response pattern across treatments (Figure 4a,b). Plant height ranged from 50 to 60.5 cm and stem diameter from 7.43 to 9.18 mm across treatments. The combined treatments (T500-BFW and T600-BFW) resulted in significantly greater plant height and stem diameter than CK. Regarding root traits, no significant differences were observed in root length among treatments (Figure 4c). In contrast, nodule number reached 13.75 and 13.83 per plant in T600 and T600-BFW, respectively, both significantly higher than that in CK (Figure 4d). These results indicate that treatments involving flame weeding at 600 °C were associated with enhanced soybean nodulation. In terms of leaf physiology, the leaf area and SPAD values of T500-BFW and T600-BFW were significantly higher than in CK (Figure 4e,f). The mean net photosynthetic rate in the CK was 1.74 μmol m^−2^ s^−1^, whereas that in T600-BFW increased to 3.90 μmol m^−2^ s^−1^, representing an increase of 124.14%, with a concurrent increase in the transpiration rate (Figure S1). Shoot N concentrations in T500-BFW and T600-BFW were 11.25 and 11.20 g kg^−1^, respectively, corresponding to increases of 7.55% and 7.07% over CK (10.46 g kg^−1^). Additionally, grain nitrogen accumulation increased from 56.8 mg in CK to 90.36 mg in T600-BFW. Among the grain quality parameters, grain moisture changed only slightly (9.14% to 9.42%). By contrast, grain protein concentration declined from 42.8% in CK to 40.15% and 41.95% in T500-BFW and T600-BFW. Crude fat content increased only marginally in T500-BFW, from 20.65% to 21.26%. Collectively, these results show that T600-BFW markedly enhanced photosynthetic performance and N/P accumulation, while the decrease in grain protein was modest, and crude fat was maintained.

### 3.5. Correlation Analysis Between Different Indicators

**Figure 5:**
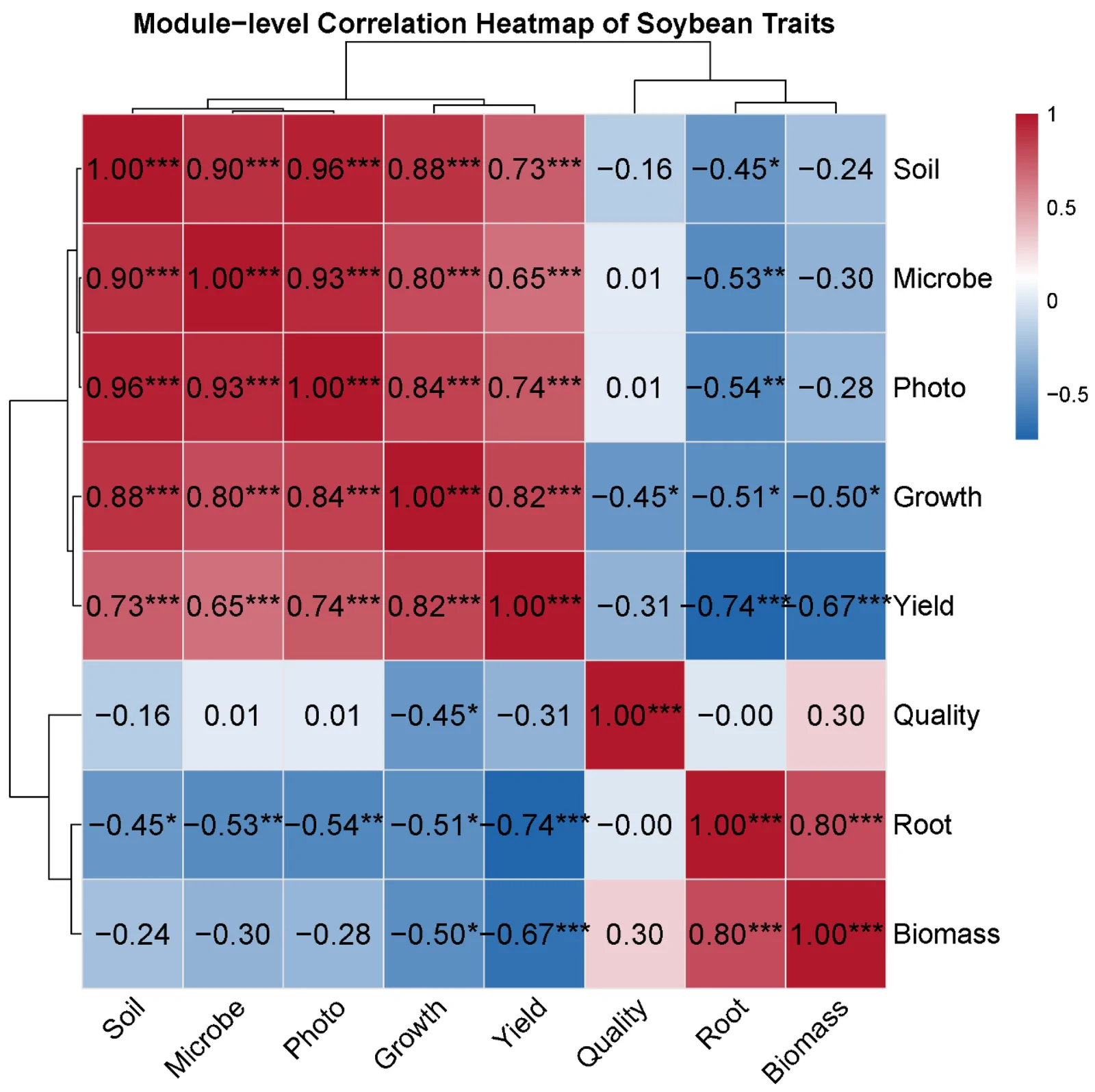
Based on the correlation analysis of 31 variables, the 31 variables were divided into 8 modules. Among them, the Soil module includes soil water content, soil temperature, soil EC, soil pH, and Eh. The Microbe module includes bacteria, actinomycetes, and fungi. The Root module includes root length, lateral root number, and nodule number. The Photosynthesis (Photo) module includes net photosynthetic rate, transpiration rate, intercellular CO_2_ concentration, and stomatal conductance. The Growth module includes leaf area, SPAD, plant height, and stem diameter. The Biomass module includes dry weights of roots, stems, leaves, and the whole plant. The Yield module includes the number of seeds per plant, 100-seed weight, yield per plant, and dry weight of seeds per plant. The Quality module includes seed moisture, protein content, crude fat content, and water-soluble protein content. “*” *p* < 0.05; “**” *p* < 0.01; “***” *p* < 0.001.

To elucidate the correlations among soil properties, soil microorganisms, soybean growth, development, yield, and quality, we performed correlation analyses based on 31 variables. These variables were categorized into eight modules, and principal component (PC1) scores for each module were used to generate the correlation heatmap (Figure 5). The results showed that the Growth, Soil, Microbe, and Photo (Photosynthesis) modules were significantly positively correlated with each other (*r* = 0.80–0.96, *p* < 0.001). The strongest correlation was observed between the Soil and Photo modules (*r* = 0.96, *p* < 0.001), suggesting a strong correlation between soil physicochemical properties and photosynthetic efficiency. In contrast, the Quality module exhibited negative correlations with the Growth and Yield modules; however, only its correlation with Growth was significant (*r* = −0.45, *p* < 0.05), whereas its correlations with the Root, Microbe, and Photo modules were near zero (*r* = 0.00–0.01, all non-significant). Furthermore, the Yield module was highly significantly positively correlated with the Soil, Microbe, and Photo modules (*r* = 0.65–0.74, *p* < 0.001), with the strongest association between the Yield and Growth modules (*r* = 0.82, *p* < 0.001).

### 3.6. Effects of Flame Weeding on Soil Microbial Communities

**Figure 6:**
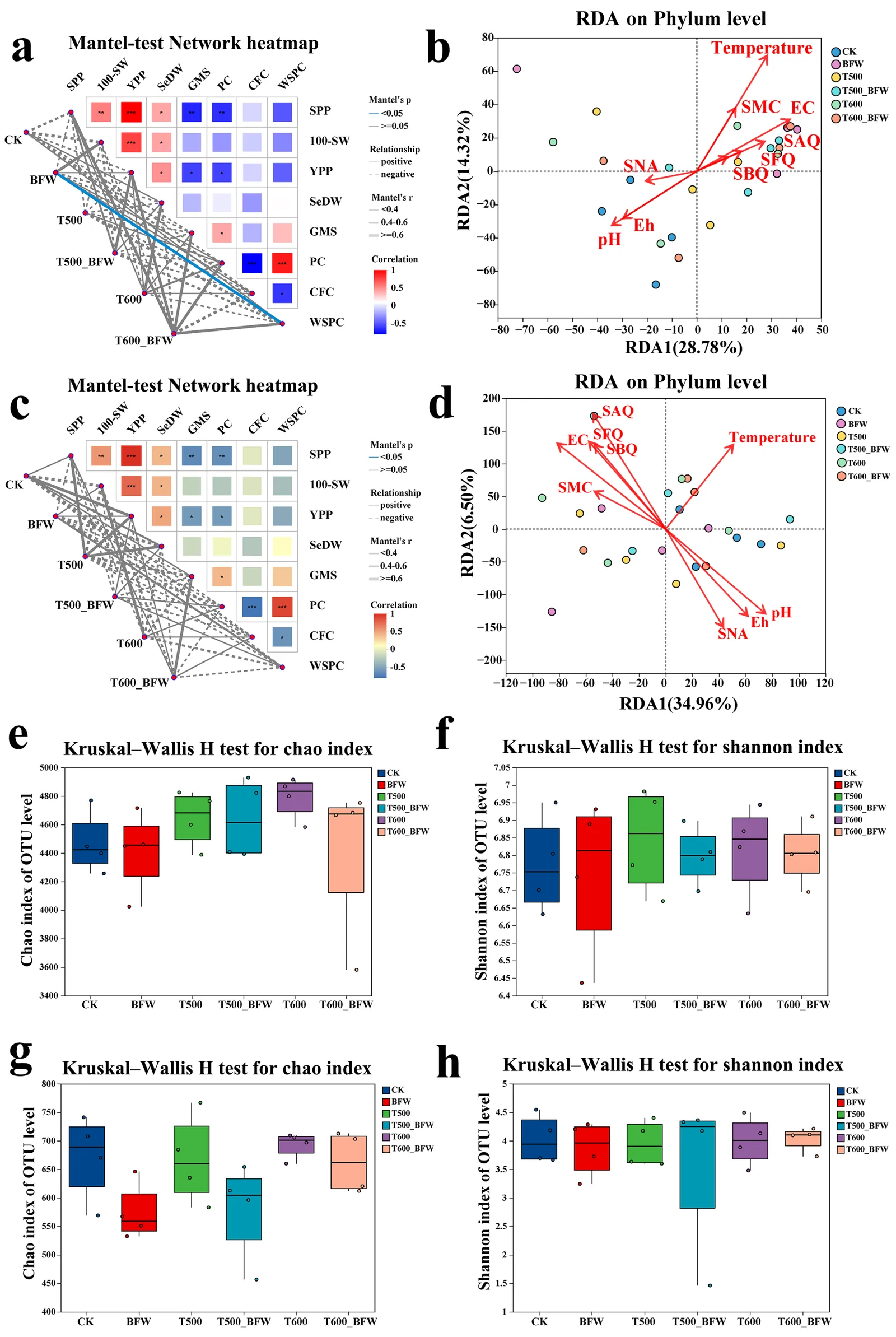
Effects of flame weeding and BFW foliar spraying on soil microbial communities. Mantel-test analysis of bacterial (**a**) and fungal (**c**) communities as well as yield and quality indicators under different treatments. RDA analysis between soil environmental factors and bacterial (**b**) and fungal (**d**) community structures. (**e**,**f**) Bacterial richness (Chao1) and diversity (Shannon) under different treatments. (**g**,**h**) Fungal richness (Chao1) and diversity (Shannon) under different treatments. “*” *p* < 0.05; “**” *p* < 0.01; “***” *p* < 0.001.

To explore whether flame weeding and BFW application affect soybean growth, development, yield, and quality through modulating soil microbial communities, 16S and ITS rRNA gene amplicon sequencing was performed on rhizosphere soil samples from the different treatments (Figure 6). T600 enhanced bacterial richness, as indicated by a higher Chao1 index than that of CK, whereas bacterial diversity, as indicated by the Shannon index, did not differ significantly between the two treatments (Figure 6e,f). For fungal communities, BFW and T500-BFW resulted in a marked decrease in fungal richness compared with the control, while no significant difference was detected in fungal diversity (Shannon index) (Figure 6g,h). Furthermore, correlation analyses between microbial community data and yield/quality traits across treatments revealed that for bacteria, only BFW exhibited a significant positive association with soybean water-soluble protein content, whereas no significant associations were detected between other treatments and yield/quality traits (Figure 6a). Bacterial communities in the CK and T500 showed negative correlations with yield-related traits (seeds per plant, 100-seed weight, and yield per plant). T500-BFW and T600-BFW displayed positive correlations with yield per plant. RDA analysis revealed that soil temperature, EC, and soil pH under different treatments were the main factors driving the variation of bacterial communities (Figure 6b). For fungi, the correlations between fungal community composition and yield/quality traits did not reach statistical significance (Figure 6c). RDA analysis revealed that EC and soil pH under different treatment were the main factors driving the variation of fungal communities (Figure 6d). Analysis of the microbial community composition revealed that at the phylum level, the bacterial community was dominated by *Pseudomonadota*, *Actinomycetota*, *Chloroflexota*, *Acidobacteriota*, and *Bacillota*, together accounting for 70–75% of the total relative abundance (Figure S3). *Pseudomonadota* was most abundant in CK (26%) but decreased following BFW application. In contrast, *Acidobacteriota* and *Bacillota* showed relatively higher proportions in BFW-treated soils. *Actinomycetota* remained relatively stable across treatments (14–17%). Fungal communities were predominantly composed of *Ascomycota* and *Basidiomycota* across all treatments. *Ascomycota* was the most abundant phylum, accounting for 70–82% of the total community. Its relative abundance decreased in the BFW treatment but remained higher in the T500, T500-BFW, T600, and T600-BFW treatments than in BFW alone. In contrast, *Basidiomycota* increased markedly under BFW treatment and remained relatively high in T600 and T600-BFW soils. *Chytridiomycota*, *Mortierellomycota*, and other fungal taxa accounted for only minor proportions.

### 3.7. PLS-PM Model for Different Indicators of Soybeans

**Figure 7:**
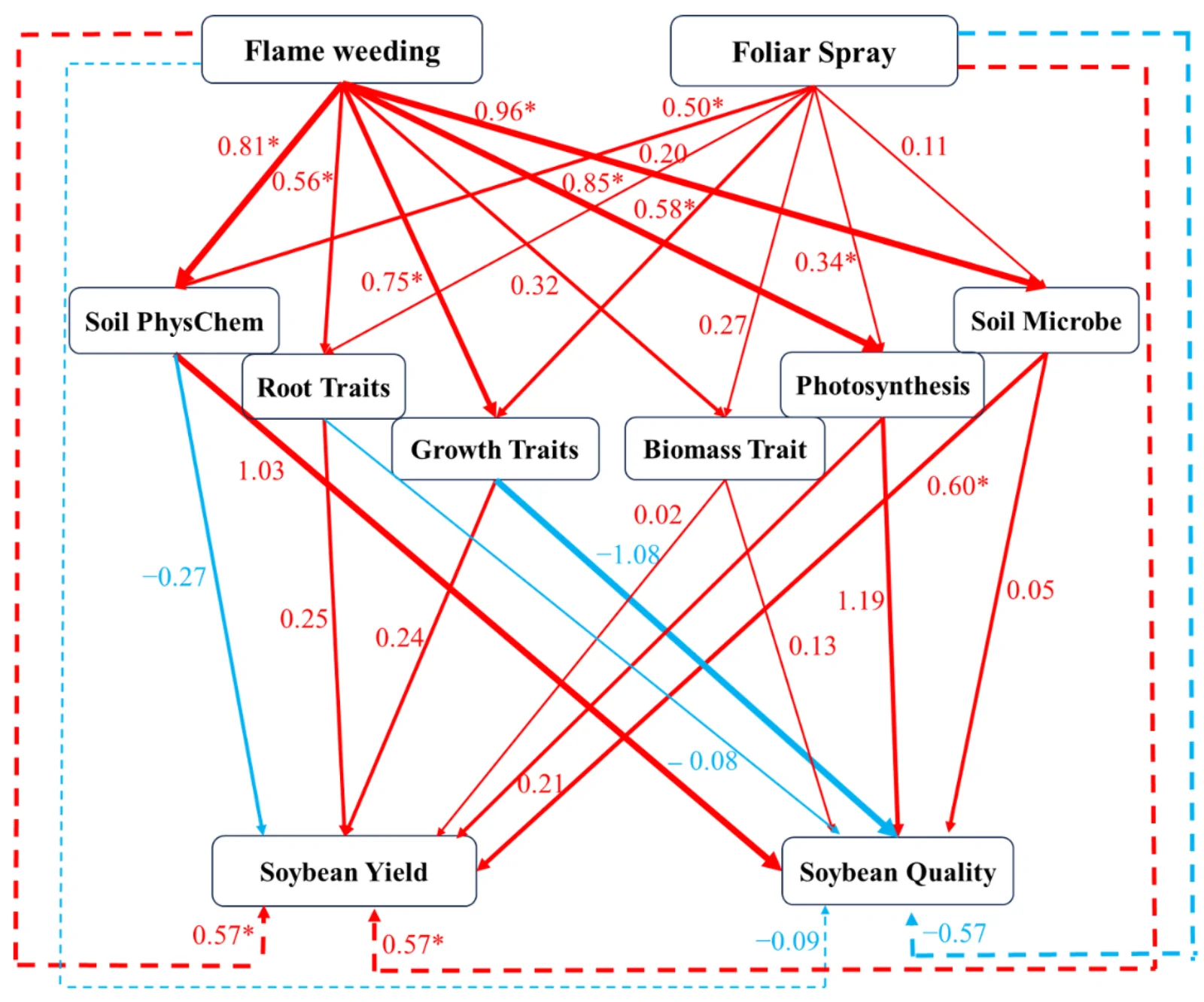
The partial least squares path modeling (PLS-PM) was employed to investigate the effects of flame weeding and foliar spraying of BFW. The standardized path coefficients are indicated next to the arrows, with the width of the arrows being proportional to the strength of the association. Red arrows represent positive relationships, while blue arrows denote negative relationships. Solid arrows mean direct effect, whereas dashed arrows mean indirect effect. The quality of the PLS-PM model is assessed by examining the goodness-of-fit index (GOF), which for this model is 0.7407. The data utilized in the model pertain to the harvest stage. “*” denote significant effect (*p* < 0.05).

To elucidate the relationships of flame-weeding temperature and BFW foliar spraying on soybean traits, a three-tiered partial least squares path model (PLS-PM) was constructed (Figure 7). The model achieved a goodness-of-fit coefficient of 0.7407. Flame-weeding temperature showed positive direct path coefficients for all endogenous constructs (path coefficients = 0.32–0.96). In particular, its paths to soil physicochemical properties, the soil microbial community, root traits, growth traits, and photosynthesis were significant. In contrast, the foliar spraying showed relatively lower path coefficients, ranging from 0.11 to 0.58; nevertheless, it still demonstrated significant positive effects on growth-related traits and photosynthesis. With respect to yield and quality, flame weeding contributed positively and indirectly to soybean yield, with a pronounced promotional effect on yield. Meanwhile, foliar spraying of BFW had a significant indirect positive impact on soybean yield (path coefficient is 0.57), albeit with a certain negative influence on quality attributes. Among the second-tier constructs, only the soil microbial community had a significant positive direct path to soybean yield. Among the estimated path coefficients, several exceeded 1 or were below −1, which may be attributable to strong multicollinearity among the relevant variables.

## 4. Discussion

### 4.1. Optimization of Flame-Weeding Temperature and Its Ecological Consequences

Flame weeding represents an environmentally friendly approach for weed control and straw residue management while also serving as a viable solution to mitigate the air pollution issues associated with conventional straw burning. Previous studies primarily focused on the timing of flame weeding and the speed of the flaming process in applied research [34,35,36,37,38]. However, the equipment and application methods employed across different studies have varied considerably. Flame weeding conducted at different speeds has revealed that except at a speed of 2 km h^−1^, some weeds survived after flame treatment at speeds of 4–8 km h^−1^. Flaming at 2 km h^−1^ reduced the number and fresh weight of weed seedlings, yet significant regrowth occurred subsequently [35]. There are still few studies on the processing and utilization of carbon materials after flame weeding. In this study, to evaluate whether flame-weeding technology can avoid the air pollution caused by traditional straw burning, we carried out a preliminary field experiment on flame weeding (Figure 1). We selected a flame-weeding temperature range of 500–600 °C based on the results of preliminary experiments conducted at 300–800 °C. The selection is mainly based on two aspects. On the one hand, flame-weeding temperatures of 500–600 °C can significantly increase the carbonization rate of weeds and greatly reduce the PM2.5 concentration. Although the CO_2_ concentration at 700–800 °C is lower than that at 500–600 °C, the existing literature indicates that as the pyrolysis temperature rises, more carbon will be lost in the form of gas. Meanwhile, higher temperatures also mean greater energy consumption and increased costs. Taking the above considerations together, we conclude that 500–600 °C is a suitable temperature range for field-scale application [39]. In the term of soil, we found that flame weeding causes short-term physical and chemical disturbances, which, in turn, lead to longer-lasting changes in some soil chemical and biological properties. The increase in soil electrical conductivity, pH fluctuations, and reduction in soil nematode abundance we observed after flame weeding are largely consistent with previous studies [40]. Furthermore, we found that there were no significant differences in soil nitrogen contents after flame weeding, indicating that short-term flame weeding was insufficient to release immobilized nitrogen elements in the soil (Figure S2). However, the soil phosphorus and organic matter content increased significantly. The soil organic matter content of T500 was markedly higher than that in the control and T600. This may because more organic matter of T600 was lost in the form of gas. Therefore, we speculate that the increase in soil EC was caused by the release of organic matter from weed residues and part of the elements into the soil after flame weeding. In soil, nematodes often have dual effects on plant growth performance. On the one hand, when nematodes pierce and suck plant roots, they can promote the colonization of plant growth-promoting bacteria in the soil on root tissues and activate plants’ defense mechanisms against the environment [41,42]. On the other hand, soil nematodes have more prominent negative impacts. Nematodes may cause the death of plant roots by piercing and sucking them. In addition, the wounds caused by piercing and sucking can indirectly trigger diseases such as wilt [43]. Although flame weeding cannot completely eliminate soil nematodes, its inhibitory effect on nematodes leads to further long-term effects (Figure 2).

### 4.2. Combined Application of Flame Weeding and BFW Enhances Soybean Yield

Flame weeding and BFW application, both recognized as measures that promote agricultural production, were observed in field trials at two locations to significantly enhance soybean yield when applied in combination (Figure 3). An intriguing finding is that although the final yield performance was similar between the two locations, the specific yield components differed. In the first season, the yield increase was primarily manifested in the 100-seed weight, whereas in the second season, the increase was more pronounced in pods per plant and seeds per plant. This indicates that the yield composition of soybean is environment-dependent. This has also been observed in other studies [44]. No significant interaction between flame weeding and BFW foliar spraying was observed for yield indicators in this study (Table S1). This may because flame weeding was only applied at the pre-sowing stage in this experiment, while foliar spraying was carried out four times after sowing, resulting in poor temporal synergy between the two practices. In addition, the promoting effects of flame weeding on soybean dry weight and pod weight found in this study are basically consistent with the conclusions of previous studies about biochar application [30,45]. This means that flame weeding can achieve agronomic effects similar to the soil application of biochar.

### 4.3. Physiological and Microbial Basis Underlying Yield Enhancement

The yield-promoting effects of flame weeding and BFW foliar spraying were accompanied by coordinated changes in soybean photosynthetic performance, root-associated traits, nutrient acquisition, and rhizosphere microbial communities. Foliar spraying of BFW alone had relatively limited effects on improving soybean yield, plant height, stem diameter, and other traits, but it significantly improved photosynthesis, leaf area, and SPAD. This is basically consistent with previous studies on biochar application to soil [46,47]. BFW foliar spraying may primarily act on leaf physiology, enhancing photosynthesis and thereby promoting nutrient accumulation. In addition, previous studies have demonstrated that foliar application of biochar confers stress tolerance [33,48]. In the study, although the yield data in the first season were lower than those in the second season, the yield-enhancing effect of T600-BFW was more pronounced in the first season (Figure 3). This may be attributed to the effect of BFW foliar spraying to enhance the tolerance of soybean under tropical high-temperature conditions in the summer, thereby preventing yield reduction. Correlation analysis indicated that enhanced growth and photosynthetic performance were generally associated with higher yield, whereas the negative direct coefficients obtained from the PLS-PM suggest that their effects on yield were not simply linear (Figure 6 and Figure 7). Instead, enhanced photosynthetic capacity may primarily increase vegetative biomass and resource acquisition, with the eventual yield response depending on assimilate partitioning toward reproductive organs. The increase in nodule number, together with enhanced N and P accumulation, suggests that the yield response was accompanied by improved nutrient acquisition and/or redistribution rather than being driven solely by increased photosynthetic activity (Figure 4 and Figure S1).

In terms of microorganisms, we observed that flame weeding and BFW treatments exerted certain effects on the microbial community composition, and the root nodule number was significantly increased under T600-BFW (Figure 4 and Figure 6). Previous studies have shown that biochar can serve as a growth carrier for rhizobia and promote root nodule formation in peat [49]. Accordingly, we speculate that flame weeding or BFW treatment may affect the growth of rhizobia in soil. Moreover, the PLS-PM model was constructed using microbial data obtained by the dilution plating method (Figure 7). We found that only soil microbial indicators exerted significant positive effects on yield. Although microbial diversity analysis revealed no significant differences in microbial abundance among treatments, shifts in the abundance or activity of certain microorganisms may still occur in the soil. Therefore, this could be pursued as a direction for further research. Microbial response to flame weeding was likely mediated, at least in part, by treatment-induced changes in the soil physicochemical environment, particularly EC and pH.

In summary, we speculate that flame weeding primarily acted through the soil–microbe–root to promote soybean yield, whereas BFW foliar spraying primarily enhanced leaf physiological performance; their combined agronomic benefits converged on yield formation through different, partly independent pathways.

## 5. Conclusions

This study demonstrates that flame weeding can be extended beyond non-chemical weed control to support the reutilization of carbonized field residues in soybean production. Under the field conditions evaluated in this study, a flame-weeding temperature of 600 °C combined with foliar application of 300-fold diluted BFW (T600-BFW) was the best-performing treatment. BFW was applied at the V4, R2, R4, and R6 growth stages. Pre-sowing flame weeding mainly modified the soil environment, including EC, pH, soil phosphorus, nematode abundance, and rhizosphere microbial composition, whereas foliar BFW application was more closely associated with improved leaf physiological performance and yield formation. Therefore, for soybean production under conditions comparable to those of the present study, we recommend pre-sowing flame weeding at 600 °C followed by four applications of 300-fold diluted BFW during vegetative growth, flowering, pod formation, and grain filling. The 500–600 °C range may be considered operationally feasible when both carbonization efficiency and particulate emissions are taken into account; however, 600 °C achieved the greatest agronomic benefit when combined with BFW in this study.

Across two field seasons, both flame weeding and BFW foliar spraying improved soybean growth and yield, with T600-BFW consistently producing the highest yield per plant and increasing yield by 73.32% and 32.43% relative to the control. These yield responses were accompanied by enhanced photosynthetic performance, greater biomass accumulation, increased nodulation, and improved N and P acquisition. Meanwhile, flame weeding and BFW application reshaped rhizosphere microbial communities, and variations in bacterial and fungal communities were closely associated with changes in soil EC, pH, and temperature. The PLS-PM model further indicated that flame weeding primarily influenced soybean production through soil-, root-, photosynthesis-, growth-, and microorganism-related pathways, whereas BFW foliar spraying exerted a strong indirect effect on yield. The significant positive path from the microbial module to yield further highlights the potential contribution of rhizosphere microbial regulation to crop productivity. Although BFW application was associated with improved photosynthetic performance and nutrient accumulation, the uptake, translocation, and physiological action of BFW-derived particles or mineral components after foliar deposition were not directly quantified. Future studies should combine multi-site and multi-year trials with measurements of foliar uptake, nutrient transport, chlorophyll fluorescence, root exudation, nitrogen fixation, soil nutrient availability, and microbial functional genes. These efforts will be necessary to validate the proposed mechanisms and establish robust application guidelines for field-scale use.

Taken together, our results suggest that flame weeding and BFW reutilization contribute to soybean productivity through partly independent but complementary pathways: flame weeding primarily modifies the soil–microbe–root, whereas BFW foliar spraying mainly enhances leaf physiological performance. Their combined use therefore provides a promising approach for integrating weed management, biomass residue reutilization, and yield improvement.

## Figures and Tables

**Table 1:** Basic properties of biochar from flame weeding.

Sample	pH	Temperature (Pyrolysis)	TN (g kg^−1^)	TP (g kg^−1^)	Elements (Wt%)					
					C	O	Si	K	Ca	Mg
BFW-500 °C	5.96 ± 0.04	500 °C	1.27 ± 0.23	2.55 ± 0.48	35.99 ± 12.62	39.02 ± 8.40	17.35 ± 2.31	3.21 ± 0.13	0.65 ± 0.19	0.66 ± 0.08
BFW-600 °C	5.81 ± 0.04	600 °C	1.51 ± 0.04	2.07 ± 0.10	33.99 ± 12.24	41.11 ± 2.43	12.52 ± 2.09	3.05 ± 0.40	0.66 ± 0.28	0.93 ± 0.66

Note: the BFW used for foliar spraying was prepared from carbonized weed residues collected after the 500 °C flame-weeding treatment.

**Table 2:** Experimental design of integrated flame weeding and BFW foliar spraying.

Treatment	Flame Weeding	Foliar Spraying
CK	No	No
BFW	No	BFW
T500	500 °C	No
T500-BFW	500 °C	BFW
T600	600 °C	No
T600-BFW	600 °C	BFW

Note: the BFW used for foliar spraying was prepared from carbonized weed residues collected after the 500 °C flame-weeding treatment.

## Data Availability

The raw data supporting the conclusions of this article will be made available by the authors on request.

## Supplementary Materials

The following supporting information can be downloaded at: https://www.mdpi.com/article/10.3390/plants15203132/s1.
