# Supplementary material for: Integrated Flame Weeding and Biochar Reutilization Enhance Soybean Yield by Promoting Soil Microbial Abundance
Source: Plants (Basel). 2026 Oct 14;15(20):3132. doi: 10.3390/plants15203132 (PMC13708631; doi:10.3390/plants15203132)
Supplement: Supplementary file 1 [file plants-15-03132-s001.zip › plants-4595117-supplementary.pdf]

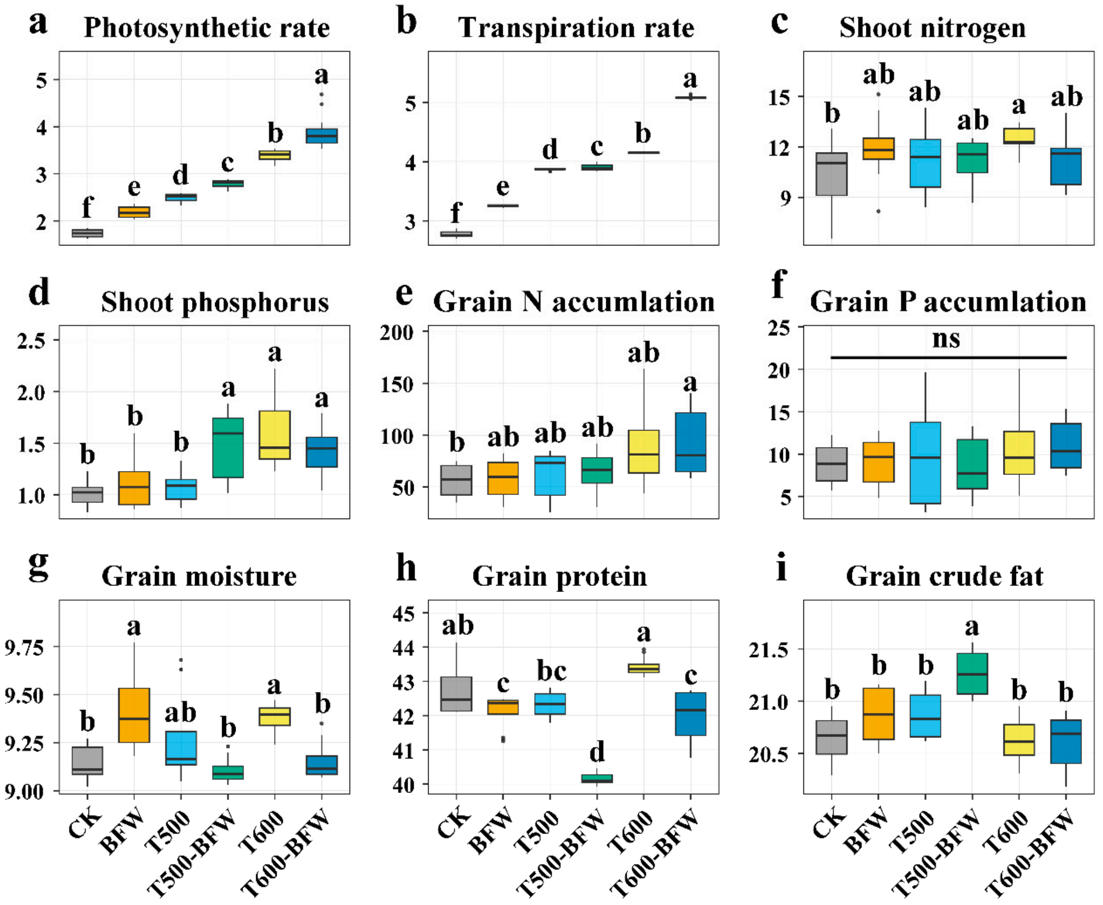

Figure S1. Effects of flame weeding and biochar from flame weeding (BFW) foliar spraying on soybean leaf gas-exchange parameters, nutrient status, and grain quality traits. Boxplots show **(a)** photosynthetic rate, **(b)** transpiration rate, **(c)** shoot nitrogen content, **(d)** shoot phosphorus content, **(e)** grain nitrogen accumulation, **(f)** grain phosphorus accumulation, **(g)** grain moisture content, **(h)** grain protein content, and **(i)** grain crude fat content under CK, BFW, T500, T500-BFW, T600, and T600-BFW treatments. The central line indicates the median; boxes represent the interquartile range; whiskers represent the data range excluding outliers; and dots indicate outliers. Different lowercase letters indicate significant differences among treatments (P < 0.05), whereas "ns" indicates no significant difference.

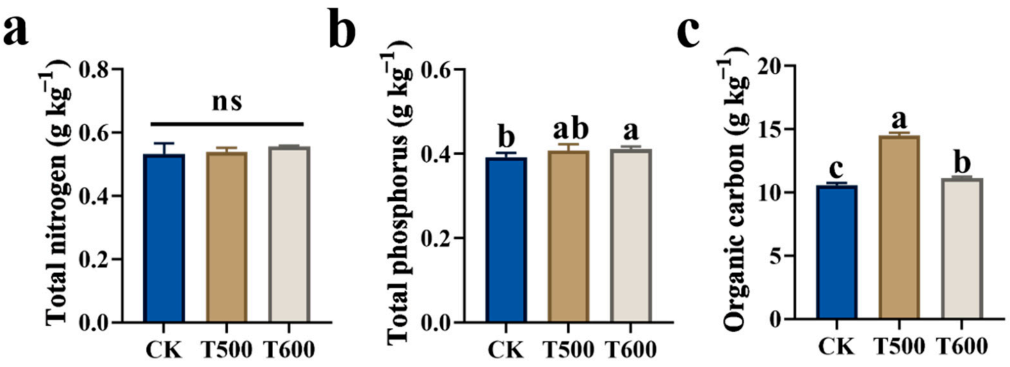

Figure S2. Soil nutrient content under different treatments at the harvest stage. (a) Total nitrogen, (b) total phosphorus, (c) organic carbon content of soil under different flame weeding. Means ± SD with different letters in the figures indicate significant differences according to the Tukey's multiple range test ($P < 0.05$) among all treatments. ns means not significant.

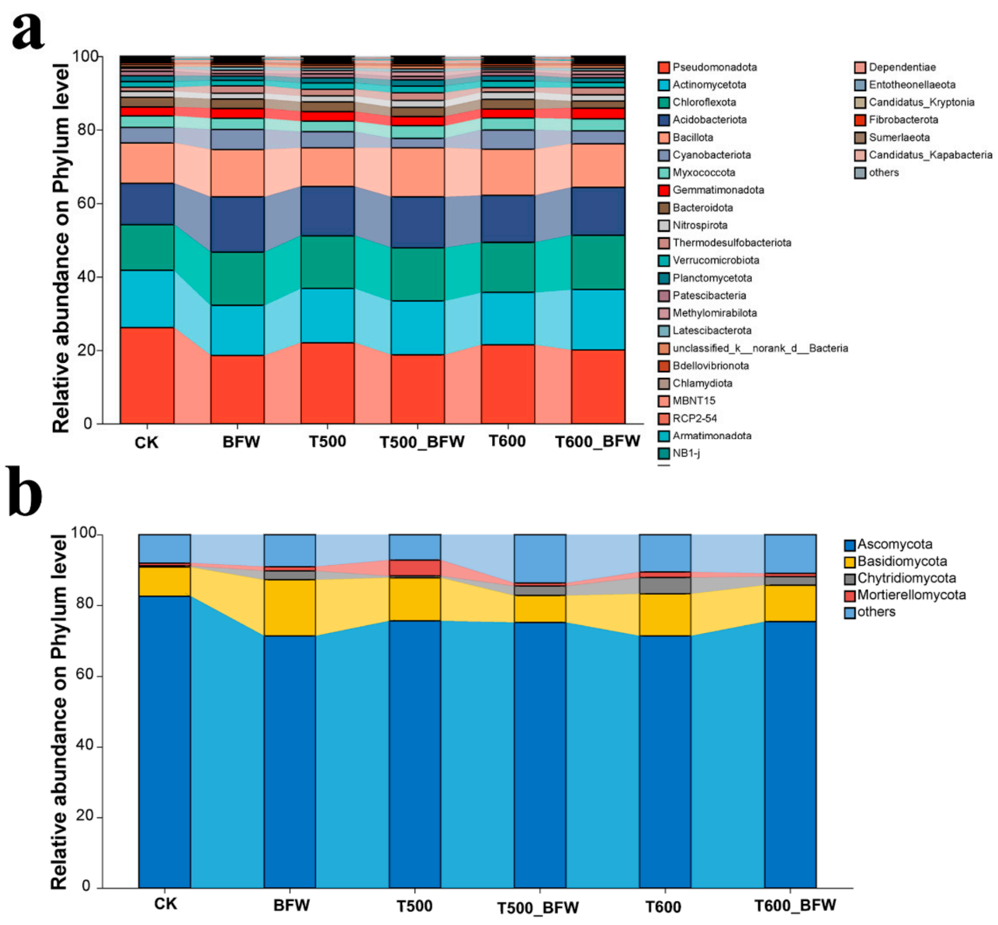

Figure S3. Relative abundances of soil bacterial (a) and fungal (b) communities at the phylum level under different treatments (CK, BFW, T500, T500_BFW, T600, and T600_BFW). Taxa with

low relative abundance were grouped as “others”.

Table S1. Tests of effects from ANOVA for flame weeding, BFW foliar spraying, and their interaction on yield in the first and second seasons.

Season	Source	Number of parameters	Df	SS	F-value	P-value
First	FW	2	2	78.10203	6.9827	0.0057*
	BFW	1	1	125.40082	22.4229	0.0002*
	FW*BFW	2	2	21.42443	1.9154	0.1761
Second	FW	2	2	46.05627	3.2046	0.0564
	BFW	1	1	178.01018	24.7718	<0.0001*
	FW*BFW	2	2	2.33221	0.1623	0.8510

Note: FW represents flame weeding; BFW represents biochar from flame weeding.
